# *In Silico* Whole Genome Association Scan for Murine Prepulse Inhibition

**DOI:** 10.1371/journal.pone.0005246

**Published:** 2009-04-16

**Authors:** Bradley Todd Webb, Joseph L. McClay, Cristina Vargas-Irwin, Timothy P. York, Edwin J. C. G. van den Oord

**Affiliations:** 1 Center for Biomarker Research and Personalized Medicine, Virginia Commonwealth University, Richmond, Virginia, United States of America; 2 Department of Pharmacy, Virginia Commonwealth University, Richmond, Virginia, United States of America; 3 Department of Human Genetics, Virginia Commonwealth University, Richmond, Virginia, United States of America; 4 Virginia Institute for Psychiatric and Behavioral Genetics, Virginia Commonwealth University, Richmond, Virginia, United States of America; Ohio State University Medical Center, United States of America

## Abstract

**Background:**

The complex trait of prepulse inhibition (PPI) is a sensory gating measure related to schizophrenia and can be measured in mice. Large-scale public repositories of inbred mouse strain genotypes and phenotypes such as PPI can be used to detect Quantitative Trait Loci (QTLs) *in silico*. However, the method has been criticized for issues including insufficient number of strains, not controlling for false discoveries, the complex haplotype structure of inbred mice, and failing to account for genotypic and phenotypic subgroups.

**Methodology/Principal Findings:**

We have implemented a method that addresses these issues by incorporating phylogenetic analyses, multilevel regression with mixed effects, and false discovery rate (FDR) control. A genome-wide scan for PPI was conducted using over 17,000 single nucleotide polymorphisms (SNPs) in 37 strains phenotyped. Eighty-nine SNPs were significant at a false discovery rate (FDR) of 5%. After accounting for long-range linkage disequilibrium, we found 3 independent QTLs located on murine chromosomes 1 and 13. One of the PPI positives corresponds to a region of human chromosome 6p which includes *DTNBP1*, a gene implicated in schizophrenia. Another region includes the gene *Tsn* which alters PPI when knocked out. These genes also appear to have correlated expression with PPI.

**Conclusions/Significance:**

These results support the usefulness of using an improved *in silico* mapping method to identify QTLs for complex traits such as PPI which can be then be used for to help identify loci influencing schizophrenia in humans.

## Introduction

Traditional approaches for mapping quantitative trait loci (QTLs) in mice usually involve crossing two strains that differ in a trait of interest, followed by phenotyping and genotyping a large number of the resulting progeny. The chromosomal regions identified with this approach are large, typically 20–40 cM [1], and further work is therefore needed to pinpoint the specific gene(s) and causal mutation(s) responsible for the QTL effect. The whole process is expensive and may require many years of study. However, databases have recently been created that contain data from large scale genotyping projects involving many common inbred mouse strains. Combining this data with phenotypic information on the same strains creates the opportunity to map QTLs “*in silico*” [2]. Since all mice from an inbred strain are genetically identical and homozygous, genotyping need only occur once and their haplotypes can be derived unambiguously from their genotypes. Once the phenotype is known one can 1) group the mice from strains with similar genotypes and then 2) test for the phenotypic differences between the mice with the different genotypes. By repeating this for a genome-wide panel of markers, a whole-genome scan can be performed *in silico* for detecting haplotypes that harbor variants influencing the trait.

There seems little doubt that *in silico* scans are useful to detect highly penetrant mutations [3]–[5] and a number of successful examples can be found in the literature [6]–[8]. However, the utility of this method for finding QTLs for complex traits is more controversial. Some criticisms involve the specific execution of the method, such as the use of a very small number of inbred strains, or insufficient control of false discoveries due to multiple testing problems [9], [10]. In principle, these criticisms can be easily addressed, by increasing the number of strains in the analysis or using new and more powerful methods to control false discoveries [11]. Other criticisms may be more fundamental, such as difficulties arising from the complex haplotype structure of inbred mice or the risk of false discoveries due to the presence of genotypic and phenotypic subgroups of mouse strains [12]. Other fast and cost-effective *in silico* methods exist, such as using a panel of recombinant inbred lines derived from only 2 parental inbred lines [13]–[15] that are much less affected by these phenomena. However, it may be premature to discard *in silico* mapping of QTLs for complex traits using common inbred strains. The method is new and at least some of the criticisms may be addressed by further developing our analytical strategies.

For example, the lack of randomness in breeding histories of inbred strains in combination with the fact that subgroups may also differ phenotypically can create spurious associations. That is, all genetic differences between the subgroups will tend to be associated with the phenotype and there would be no possibility to distinguish true and spurious associations. In human studies, an analogous issue is population stratification, which is a great concern. In samples containing subjects with multipleancestries, this must be addressed using appropriate statistical controls [16]. Otherwise, tens of thousands or markers will appear significant in the genome-wise association studies using up to one million genetic markers. Approaches to control for stratification include using of self report of ancestry or genetically derived principle components in the analysis. For studies using inbred mouse lines, a cladogram which is a hierarchical grouping based on phylogenetic analysis of strain relatedness can be created to subdivide inbred strains into more genetically homogenous subgroups. By testing whether or not haplotypes are associated with the phenotypes *within* these cladistic subgroups which are akin to branches in the tree, we reduce the risk of false positives. This is because genetic variation is now related to deviations from the subgroup mean, so that phenotypic differences between strains are no longer necessarily associated with genetic differences between strains.

If we assume that the methodological problems can be addressed, *in silico* scans do have a number of potential advantages. First, since the average ancestral segment length among classical inbred strains has been estimated to be 1.0–1.5 megabases (Mb) in size, the resolution is relatively good in comparison to traditional QTL mapping methods. Second, the costs for many common inbred mouse lines are relatively low in comparison to recombinant inbred lines. Third, the amount of phenotypic and genotypic information on common inbred strains is increasing rapidly. Examples of freely available repositories such as the Mouse Phenome Database (MPD - [17]) and WebQTL continue to grow. These resources include not only strain phenotypes but also genotypes from large scale projects that have recently been completed (http://www.well.ox.ac.uk/mouse/INBREDS/) and others in progress. The availability of all this information has the potential to produce novel results with the only cost being analysis time. Finally, the presence of multiple founder lines as well as wild derived inbred strains can be advantageous. First, there is more genetic and phenotypic diversity when many strains are used. Therefore the potential to detect more causal variants is increased. In mapping using F2 crosses of only two strains, much of the variation that is present in the population from which the two lines were drawn is excluded and not detectable. Although the use of multiple founder lines introduces more alleles and decreases relative effect size, the process is much more analogous to human association mapping. Therefore, the results may also be more generalizable across lines and perhaps species. This is important because the generally accepted eventual goal of using model organisms is to generalize the knowledge to humans. Results such as these can be used in cross species data integration[18] which can lead to the identification of novel associations in humans[19].

Although clearly of potential utility, *in silico* scans alone will probably not be able to identify the actual causal variants. Instead they may better be viewed as part of a fast and inexpensive method to identify and prioritize complex-trait candidate genes without requiring the construction of (sub)congenic mouse strains [8]. The likely outcome of an *in silico* scan is a number of small chromosomal regions that contain causal variants. Existing databases can then be used to identify the candidate genes in the regions and look for corroborating evidence. Furthermore, other “omic” platforms (e.g. expression arrays) could be use to further reduce the list of candidate genes and refine the region [20]

In this study we performed an *in silico* scan using phenotypic data generated by Willott and colleagues [21] for the complex trait prepulse inhibition (PPI), a sensory gating measure thought to be related to schizophrenia. A recent review cited 13 different studies that found PPI deficits in schizophrenic patients [22]. PPI is also variable and heritable in humans with and without psychiatric diagnoses [23], [24] and in model organisms. Deficits in PPI can be induced pharmacologically and reversed with antipsychotics [25]. PPI has also been the subject of phenotypic characterization[26], [27] and QTL mapping efforts in rodents [28]–[35]. QTLs identified *in silico* were compared against evidence from a variety of sources including previous mouse PPI QTLs, meta-analysis of human schizophrenia genome scans, and microarray experiments in an attempt to find convergent or consistent patterns of evidence.

## Results

### *In silico* scan

**Figure 1:**
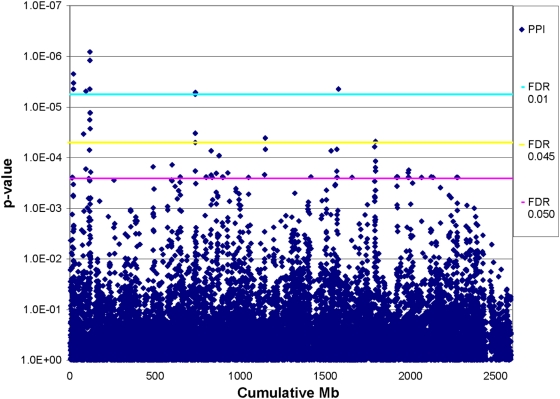
Plot of p-values from PPI scan across mouse genome with corresponding FDR thresholds.

Our base model was a 2-level model where mice were nested in strains with sex and clade membership included as covariates. SNPs were added to this base model and tests performed to examine whether this significantly improved model fit. Figure 1 plots the p-values for all SNPs across the mouse genome. The conservative “lowest slope” method (Hsueh et al., 2003) estimated the proportion of true null hypotheses to be 0.991017. Using this estimate, we found 89 significant SNPs when the FDR was controlled at the 0.05 level. Because of the large number of tests, this means that the estimated proportion of false discoveries among the 89 significant tests was 5%. The number of significant SNPs dropped noticeably from 89 to 20 when the FDR was controlled at the 0.045 rather than 0.05 level, which corresponded with a threshold p-value of 5.0e-5. We focused these SNPs in order to have tractable number of results to interpret. The full list of results satisfying a FDR of 5% are contained in Table S1.

The top 20 significant SNPs map to 8 regions with 5 isolated SNPs, 2 clusters of 3 to 4 SNPs, and one cluster of 8 SNPs. Details are contained in Table 1. Examination of linkage disequilibrium (LD) using *r* ^2^ between the top 20 markers revealed meaningful LD (<1% for genome-wide marker-marker *r* ^2^) between many of the markers pairs including those on different chromosomes. Table 2 contains these results and shows that within the set of 20 there are three sets of mirror markers containing 7, 3, and 2 markers. However, they all contain at least one marker from the cluster of positive markers on chromosome 1 between 115.9–118.9 Mb. SNP and gene positions are based on the May 2004 assembly (Build33) of the mouse genome at the UCSC Genome Browser [36]. After examining the *r* ^2^ for each of the top 20 markers with every other marker in the genome and the 2 marker association results within each mirror (data not shown), we believe that the most parsimonious explanation of the pattern of results is that the cluster on chromosome 1 is origin of the mirrors. After accounting for mirrors that reflect the cluster of significant markers on chromosome 1, only 2 additional independent signals remain and are rs3698264 (chromosome 1, 79.9 Mb, index 575) and rs3724682 (chromosome 13, 46.8 Mb, index 12594).

**Table 1:** Chromosomal band, megabase (Mbp) location, and P-value of SNPs that are significant when the FDR is controlled at 0.045 level.

Cluster	Marker	Index	chr	Mbp	Cytogenetic location	p-value
1	rs6404446	140	1	20.994482	1qA4	4.39E-06
1	rs3716569	141	1	21.012714	1qA4	2.21E-06
1	rs4222181	142	1	21.024671	1qA4	3.33E-06
2	rs3698264	575	1	79.865390	1qC4	3.39E-05
3	rs6268443	657	1	93.319361	1qD	4.86E-06
4	rs3022830	802	1	115.928102	1qE2	1.78E-05
4	rs3694226	811	1	117.143094	1qE2	4.39E-06
4	rs3662732	813	1	117.337845	1qE2	1.20E-06
4	rs3674655	814	1	117.379659	1qE2.3	8.14E-07
4	CEL-1_117526378	816	1	117.526378	1qE2	8.14E-07
4	rs6216134	820	1	118.236415	1qE2	1.29E-05
4	rs3719973	824	1	118.834067	1qE2	1.29E-05
4	rs13476078	825	1	118.930213	1qE2	2.67E-05
5	rs6215373	5262	5	42.914749	5qB3	3.27E-05
5	mCV22331571	5265	5	43.271643	5qB3	5.00E-05
5	rs3669254	5266	5	43.347335	5qB3	5.59E-06
5	rs3663092	5270	5	43.668813	5qB3	5.15E-06
6	rs3691954	8102	8	21.206986	8qA2	4.09E-05
7	rs6299418	11098	11	66.784499	11qB3	4.39E-06
8	rs3724682	12594	13	46.810500	13qA5	4.75E-05

Index is the marker order across the genome and is used in subsequent tables and figures instead of marker name.

**Table 2:** LD pattern of 20 SNPs FDR < = 0.045.

Marker	Index#	140	141	142	575	657	802	811	813	814	816	820	824	825	5262	5265	5266	5270	8102	11098	12594
rs6404446	140	1	1	1	0.05	0.51	0.62	1	0.77	0.77	0.77	0.51	0.51	0.77	0.62	0.51	1	1	0.51	1	0.24
rs3716569	141	1	1	1	0.06	0.51	0.62	1	0.77	0.77	0.77	0.51	0.51	0.77	0.62	0.51	1	1	0.51	1	0.24
rs4222181	142	1	1	1	0.05	0.51	0.62	1	0.77	0.77	0.77	0.51	0.51	0.77	0.62	0.51	1	1	0.38	1	0.23
rs3698264	575	0.33	0.42	0.38	1	0.09	0.1	0.05	0.08	0.06	0.06	0.09	0.09	0.08	0.08	0.09	0.05	0.06	0.01	0.05	0.1
rs6268443	657	1	1	1	0.33	1	0.82	0.51	0.66	0.66	0.66	0.67	0.67	0.37	0.51	0.67	0.62	0.51	0.51	0.51	0.47
rs3022830	802	1	1	1	0.5	1	1	0.62	0.81	0.8	0.8	0.82	0.82	0.47	0.63	0.51	0.62	0.62	0.47	0.62	0.39
rs3694226	811	1	1	1	0.33	1	1	1	0.77	0.77	0.77	0.51	0.51	0.77	0.62	0.51	1	1	0.51	1	0.24
rs3662732	813	1	1	1	0.5	1	1	1	1	1	1	0.66	0.66	0.47	0.8	0.37	0.77	0.63	0.64	0.77	0.31
rs3674655	814	1	1	1	0.33	1	1	1	1	1	1	0.66	0.66	0.58	0.8	0.37	0.77	0.77	0.77	0.77	0.31
CEL-1_117526378	816	1	1	1	0.33	1	1	1	1	1	1	0.66	0.66	0.58	0.8	0.37	0.77	0.77	0.77	0.77	0.31
rs6216134	820	1	1	1	0.33	0.82	1	1	1	1	1	1	1	0.66	0.51	0.4	0.51	0.51	0.51	0.51	0.28
rs3719973	824	1	1	1	0.33	0.82	1	1	1	1	1	1	1	0.66	0.51	0.4	0.51	0.51	0.51	0.51	0.28
rs13476078	825	1	1	1	0.5	0.74	0.76	1	0.76	0.76	0.76	1	1	1	0.46	0.37	0.77	0.78	0.22	0.77	0.15
rs6215373	5262	1	1	1	0.33	0.79	0.79	1	1	1	1	0.79	0.79	0.75	1	0.27	0.62	0.62	0.62	0.62	0.38
mCV22331571	5265	1	1	1	0.33	0.82	0.79	1	0.74	0.74	0.74	0.63	0.63	0.74	0.57	1	0.62	0.51	0.24	0.51	0.47
rs3669254	5266	1	1	1	0.35	1	1	1	1	1	1	1	1	1	1	1	1	1	0.51	1	0.27
rs3663092	5270	1	1	1	0.5	1	1	1	1	1	1	1	1	1	1	1	1	1	0.31	1	0.24
rs3691954	8102	0.71	0.72	0.71	0.2	1	0.76	0.71	0.8	1	1	1	1	0.52	1	0.68	0.71	0.7	1	0.51	0.24
rs6299418	11098	1	1	1	0.33	1	1	1	1	1	1	1	1	1	1	1	1	1	0.71	1	0.24
rs3724682	12594	1	1	1	0.4	1	1	1	1	1	1	0.77	0.77	0.69	1	1	1	1	1	1	1

*r* ^2^ is above diagonal. D′ is below diagonal.

### Support for results

The identified QTLs were compared with a variety of sources including previous mouse PPI QTLs, meta-analysis of human schizophrenia genome scans, and microarray experiments. The mouse/human chain track within the genome browser was used to compare regions homologous between mouse and human genomes [37], [38].

### Replication of previous mouse QTLs

There are previous studies attempting to map QTLs for PPI in mice. Joober and colleagues provisionally mapped PPI QTLs using recombinant congenic strains based on inbred lines C57BL/6J and A/J [29]. For auditory PPI, they initially reported 7 QTLs common across all acoustic intensities studied and an additional 25 loci linked to at least one acoustic intensity for a total of 32 provisional loci. However, the analytical methodology was criticized [39] and a more appropriate analysis showed a more modest list of significant loci which included chromosomes 2, 3, 5, 7, 11, and 16 [40]. The results from chromosome 16 have been investigated further by Petryshen [31] who performed QTL mapping by intercrossing chromosome substitution strains (CSS). The parental CSSs carried an A/J chromosome 16 on a C57BL/6J background. The 2 initial QTL intervals described by Joober and colleagues on 16 were confirmed and the interval narrowed. We do not believe our results robustly replicate any reported QTL on 16. Joober *et al.* have since expanded upon their auditory work using tactile PPI which didn't replicate their auditory PPI results[35].

PPI QTL mapping has also been performed using an F2 cross of C57BL/6 and C3H/He lines and identified a PPI locus at the *Fabp7* gene [33] on chromosome 10. We did not detect any significant markers in the region of *Fabp7*. Watanabe *et al.* also reported provisional QTLs on chromosomes 1, 3, 7, 11, and 13. However, the sizes of the linked regions were not reported and therefore any overlap with loci on chromosomes 1, 11, and 13 in the current study could not be compared directly.

Finally, Hitzemann and colleagues have attempted to map PPI QTLs using selectively bred lines from a heterogeneous stock derived from four inbred lines including C57BL/6J, DBA/2J, BALB/cJ and LP/J[34]. This effort is the most analogous to the current study due to the use of multiple founder lines. However, the study was directed at previously implicated chromosomes 3, 11, and 16. The signal we detected at rs6299418 on chromosome 11 is consistent with the interval reported by Hitzemann *et al* [34].

### Loci for human schizophrenia

We examined if the three independent *in silico* mouse PPI QTLs results mapped to the regions implicated by the [41] meta-analysis of human schizophrenia genome scans. The meta-analysis is a large study using 20 linkage scans with a total of 1,208 pedigrees and 2,945 affecteds. In the study, the genome was divided into 120 separate 30-cM bins. The top ten bins represent 8 different regions comprising 300 cM or ∼8% of the human genome. Four of the eight homologous mouse regions contained at least 1 significant SNP in our scan, when the FDR was controlled at the 0.05 level, including the signals on chromosomes 1 and 13.

**Figure 2:**
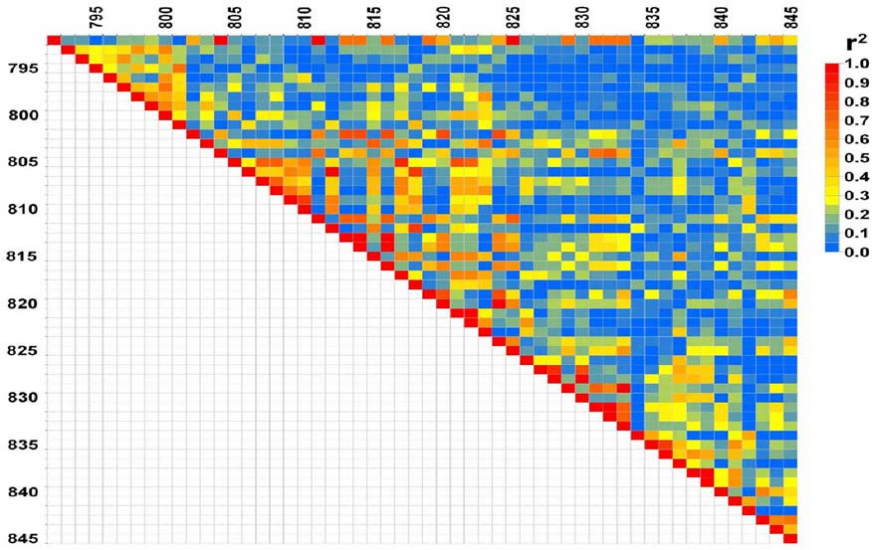
Plot of linkage disequilibrium (LD) around associated SNPs on chromosome 1. The numbers on the axes are the marker index which is the relative order of the SNPs across the genome and corresponds to the results in Table 1.

On chromosome 1 in the area surrounding marker rs3674655 (p-value 8.14×10^−7^, index814), which is homologous to human 2q14, the LD pattern shown in Figure 2 is irregular. There are no obvious places to define a boundary, even when attempting to use an arbitrary standard such as *r* ^2^ above a whole genome cut off of one percentile. By examining individual haplotypes (data not shown), we estimate the core of the association signal extends at least 3 megabases (Mb) from rs13476069 (index 802, 115.9 Mb) to rs13476078 (index825, 118.9 Mb) but may extend as much as 5.7 Mb from mCV23695506 (index792, 114.4 Mb) to rs3696498 (index 833, 120.1 Mb).

The 5.7 Mb interval includes genes *Tsn*, *Inhbb*, *Ralb*, *Epb4.1l5*, *Ptpn4*, *Sctr*, *Dbi*, and *Steap3*, several of which are good functional candidates for PPI and schizophrenia. *Ptpn4* (the protein tyrosine phosphatase, non-receptor type 4) interacts with glutamate receptors, *Grin2a* and *Grid2*. Glutamate receptors are good candidates for schizophrenia and *GRIN2A* has been the subject of human schizophrenia association studies [42]–[44]. *Sctr*, the secretin receptor gene, is also a good candidate since phencyclidine-induced impairment of PPI is partially reversed by secretin [45]. Finally, Translin (*Tsn*) is a gene known to alter PPI when knocked out in mice [46]. In addition, the TSN protein (also designated TB-RBP) interacts functionally with translin-associated factor X (TSNAX or TRAX) [47]. The human *TRAX* gene is adjacent to *DISC1*, a gene implicated in schizophrenia, and haplotypes covering both *DISC1* and *TRAX* in humans have been reported to be associated with schizophrenia [48], [49]. Several *DISC1* transcripts contain *TRAX* sequence including one that encodes a *TRAX/DISC1* fusion protein [50]. Therefore, *TSN* may interact with *DISC1*.

**Figure 3:**
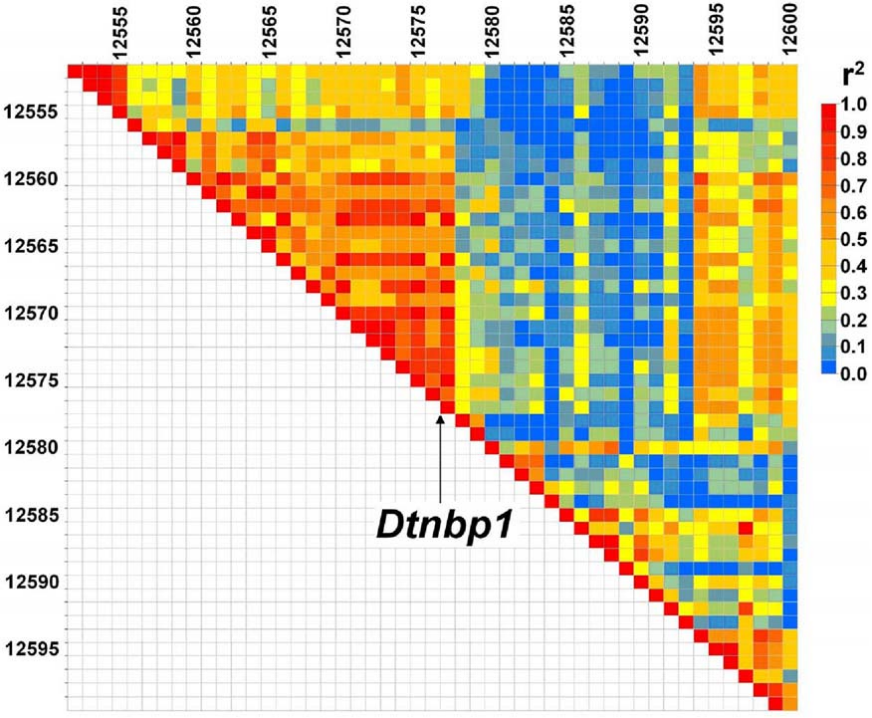
Plot of LD near associated SNPs near DTNBP1 (index12555) and rs3724682 (index12594).

In contrast to the multiple signals and inconsistent LD pattern seen on chromosome 1, the interval on 13qA5 between rs6271232–rs6244558 (minimum p-value 4.8×10−5, FDR <0.045) shown in Figure 3 presents a much more regular pattern of LD even though the interval is large (∼5.4 megabases). Interestingly, *Dtnbp1* sits in the middle of the interval. The human homolog of this gene has demonstrated multiple highly significant associations with schizophrenia, [51]–[60]. Although there is a region of relatively reduced LD in the middle of the interval, the markers flanking *Dtnbp1* are in LD with the SNPs showing association at either end. Also mapping to this interval is *Cap2*. The human homolog of this gene has been reported to show altered expression in human schizophrenic brain [61]. This interval was tentatively implicated by Joober et al. in their study of mouse PPI [29]. However, it was not one of the six chromosomes that remained after reanalysis [40].

The results for the third *in silico* QTL at rs3698264 (chromosome 1, 79.9 Mb, index 575, p-value 0.000034) appeared to be an isolated signal when only single marker analysis was considered. However, tests of sliding 2 marker windows showed additional evidence independent of rs3698264. In the 1 megabase interval surrounding but not including rs3698264, the p-values ranged from 0.045 to 0.00083. The most significant 2 marker result which includes rs3698264 is for rs3698264-rs8253473 (p-value 5.8×10^−7^) and defines an 89.5 kilobases (kb) interval containing part of the secretogranin II (*Scg2*) gene. Indeed, *Scg2* also known as chromogranin C contains rs8253473. This marker defines the end of the most associated two marker haplotype in the genome and not just the region nearby. *Scg2* is a plausible candidate for influencing PPI. Phencyclidine (PCP) modulates *Scg2* expression in rats [62], [63]. Genes responding to PCP are good candidates for schizophrenia since PCP produces effects similar to schizophrenia in humans. In model organisms, PCP creates PPI deficits that can be ameliorated with administration of atypical antipsychotics [25]. There are also positive human schizophrenia association studies with chromogranin B which is a closely related gene [64], [65].

### PPI and selected gene expression in hippocampus

Selected gene expression information was obtained via WebQTL. The data was generated by the Hippocampus Consortium on M430 arrays using hippocampus tissue and analyzed using the RMA method. Data was available for 12 of the 40 lines with PPI data. We tested for correlation between PPI and hippocampus expression of candidate genes selected from the top 3 regions. Due to the small number of lines (n = 12) with both PPI and expression information, we chose only to test microarray probesets in a limited number of genes that we had prior evidence for a relationship to schizophrenia or PPI. These genes were *Scg2*, *Dtnbp1*, *Cap2*, and *Tsn*. Details of the results are shown in Table 3. PPI was significantly correlated with gene expression for one of two probesets in *Cap2* (r = −0.6, p-value = 0.039) and approached significance using at least one probeset in *Dtnbp1* (p-value 0.1) and *Tsn* (p-value 0.085). We performed exploratory analysis looking for statistical interactions between gene expression levels and PPI using linear regression and mixed models. We observed that different probesets within the same gene such as with *Tsn* gave different results. Although this may seem inconsistent, further examination of the alignment of probes to gene revealed that different probesets for *Tsn* aligned to different populations of alternatively polyadenylated transcripts. Further analysis using univariate mixed models revealed the same pattern results across the probesets but with increased significance. A highly significant interaction was detected between *Dtnbp1* and *Scg2* (p-value = 0.00024). Although *Tsn* (mixed model p-value 0.052) and *Cap2* (mixed model p-value 0.02) are significant when considered individually and together (mixed model p-value 0.009, based on 2df), they do not contribute significantly in the presence of the *Dtnbp1*-*Scg2* interaction.

**Table 3:** Results of correlation (*r*) and linear regression (adjusted *r* ^2^) using gene expression of selected microarray probes from candidate genes near positive regions from *in silico* scan.

Gene	Probe	*r*	*adj r^2^*	p-val
*Cap2*	1450910	−0.14	−0.08	0.67
*Cap2*	1423222	−0.60	0.30	0.039
*Dtnbp1*	1431619	−0.50	0.18	0.10
*Scg2*	1450708	−0.47	0.14	0.12
*Tsn*	1448516	0.52	0.19	0.085
*Tsn*	1448515	0.40	0.08	0.19
*Tsn*	1416908	0.20	−0.05	0.53
*Tsn*	1416907	0.20	−0.06	0.53

Gene expression microarray analysis method is RMA.

## Discussion

We found 89 SNPs that were likely to have real effects on PPI (FDR<0.05). When we conservatively considered only the top 20 based on FDR and the distinct LD pattern of the inbred mouse genome, these 20 collapsed into 3 probable distinct independent regions. These 3 independently associated loci are likely to affect PPI (FDR 0.045) including a 3 to 6 megabase interval on chromosome 13 and two separate loci on chromosome 1. Next, we showed that 2 of these regions correspond to regions implicated in human linkage studies of schizophrenia. The region on chromosome 13 that is implicated by both our *in silico* PPI analyses and human linkage studies was also implicated in a provisional QTL mapping study of PPI in mice by Joober et al [29] using recombinant congenic strains. However, chromosome 13 did not remain significant when a more appropriate analysis was conducted[40]. The gene *Dtnbp1* is in the middle of this region. This is an encouraging finding as several association and expression studies suggest that the human homolog of *Dtnbp1* is one of the strongest candidates for schizophrenia [51]–[60]. In addition, the human homolog of another gene in this region, *Cap2*,is reported to show altered expression in schizophrenic brain [61].

The region on chromosome 1 that is implicated by both the *in silico* PPI analyses and human linkage studies contains the genes *Tsn and Scg2*. *Tsn* is directly implicated in mouse PPI as it is known to alter PPI when knocked out [46] and *Scg2* is PPI candidate due to multiple lines of evidence [25], [62]–[65]. Finally, we found that hippocampus expression is at least suggestively significantly related to PPI for all four genes *Dtnbp1*, *Cap2*, *Tsn*, *Scg*. Analyses of these expression data also showed a highly significant relationship between PPI and a statistical interaction between *Dtnbp1* and *Scg2*.

In sum, results suggest that the *in silico* mapping of QTLs can be improved and successfully adapted to help map loci for complex traits. That is, the obtained results were supported by converging evidence from a variety of sources including previous mouse PPI QTLs, meta-analysis of human schizophrenia genome scans, and microarray experiments. *In silico* scans have several attractive properties such as the low costs of the mice, relatively more genetic variation due to multiple ancestral strains, and public availability of genotype/phenotype information. This suggests that these scans can be a valuable addition to our method arsenal for mapping genetic variation affecting complex traits. Although the resolution is relatively good in comparison to traditional QTL mapping methods, the QTLs detected by the *in silico* methods still spanned 2–4 MB. However, we also demonstrated how public resources can be used to add weight to findings and identify specific candidates. As the amount and quality of information in public data bases increases, we would expect this ability to refine the location of relevant genetic variation to improve in parallel. Finally, as the focus in the present study was to demonstrate the usefulness of the method, we focused on genes and loci for which there is already a considerable amount of evidence in the literature. However, this does not mean that the method cannot generate novel candidates and even in our case we expect that other previously less studied genes could affect schizophrenia and are performing association studies to follow up these leads.

## Materials and Methods

### Sample and measurements

To perform an *in silico* scan, we first matched PPI data for 37 different strains to 17,757 SNPs contained in the MPD. The 37 phenotyped strains with genotype information were from a study 40 strains and represented 805 individual inbred mice with approximately 10 animals of each sex per strain [21]. The majority (∼13 k) of the SNP data came from the Wellcome-CTC Mouse Strain SNP Genotype Set (www.well.ox.ac.uk/rmott/MOUSE/INBREDS) and the remainder from a variety of other sources including dbSNP, the Jackson Lab [66], and The Scripps Research Institute [5]. The PPI variable analyzed was PPI total, which is a summary measure across three different acoustic startle frequencies (70 dB at 4, 12, and 20 kHz). Although other PPI variables were generated by Willott and colleagues, they argue that PPI total is the best measure for sensory gating. Ambiguous genotypes and heterozygotes were removed. One hundred seventy-three markers were found to be named duplicates and removed. A further 725 markers were removed from analysis because they were not polymorphic between our 37 selected strains. This left a total panel of 16,859 SNPs.

### Phylogenetic analyses

The lack of randomness in the breeding history of inbred strains in combination with the fact that strain subgroups may also differ phenotypically can create spurious associations. Our approach to minimize such spurious findings mimics “within family” based analyses often applied in human association studies to avoid similar problems due to population stratification. By testing whether a locus within a family is associated with the outcome, spurious associations are avoided because all family members come from the same subpopulation. To define “families” of inbred strains of mice we estimated the phylogenetic relationship for 480 inbred strains of mice using the APE (Analyses of Phylogenetics and Evolution) extension to the R language (results not shown). Because an extensive search is impossible with as many as 15 k SNPs we used the maximum parsimony algorithm that minimizes the number of steps, or tree length, needed to account for the differences between strains. The main groupings we observed replicated those found by Petkov et al. who constructed a “family tree” for 102 strains using 1,638 SNPs [66]. Each of the 37 strains used in the current study were assigned to one of 7 possible phylogenetic subgroups or clades from our cladistic analysis. Family trees and cladograms are not synonymous since cladistics has its own set of rules for defining family trees. Therefore, not all hierarchical arrangements of strains are cladograms since cladograms reflect similarity and not descent. Details of the strains used and clade assignment are contained in Table S2.

### Mixed/multilevel models

The tests between phenotype and genotype were performed using multilevel or mixed modeling [67], [68]. Multilevel models are particularly suitable for analyzing samples with a hierarchical or clustered structure. Clustered data are present here because multiple animals from the same strain are assessed. The inclusion of SNPs as well as subgroup membership in multilevel models is straightforward [69]. Specifically for the current study, let *o* be an overall constant, *c*_k_ the effect of phylogenetic subgroup k, *g*_j_ the effect of SNP *j*, and *r*_ijk_ a residual score of mouse *i* with genotype *j* from subgroup *k* consisting of the effects of other unlinked loci and environmental factors. The trait score *x*_ijk_ of mouse *i* with SNP *j* from subgroup *k* can then be written as: *x*_ijk_ = *o*+*c*_k_+*g*_jk_+*r*_ijk_. The statistical test will involve effect *g*_jk_. Because *g*_jk_ is the deviation from the subgroup mean it will only be significant if within the subgroup SNP *j* has an effect. Scripts were written and analyses performed in the R statistical environment. Specifically, the nlme package was used to perform mixed/multilevel model analysis. The maximum likelihood (ML) method was used instead of the default restricted maximum likelihood (REML) to be able to perform tests for fixed plus random effects in the model. Two times the difference between log-likelihoods of the model with and without genetic effect *g*_jk_ is asymptotically chi-square distributed, with the difference in estimated parameters of the nested models as the degrees of freedom. After single marker analyses were completed, haplotype analyses with multiple markers were performed to determine risk haplotype and estimate the size of the associated region.

### Control of false discoveries

In our *in silico* scan, the vast majority of the SNPs will not be associated with the dependent variables and this creates a considerable risk of false discoveries. In this article, we control the so-called false discovery rate [70], [71]. Because of the large number of tests that are performed in this study, we can interpret the FDR as the proportion of false discoveries to the total discoveries we would on average introduce into the literature through this study. Alternatively, FDR can be interpreted as the probability that a randomly selected discovery from this study is false [71]–[73].

An important advantage of the FDR in this context is that it provides a better balance between finding true effects and controlling false discoveries compared to more traditional “family-wise” methods that control the probability of finding one or more false discoveries in the whole study (e.g. the single step Bonferroni correction). The problem is that family-wise error methods control exclusively to the risk of even a single false discovery. Because this risk is high in genome-wide scans, these studies will be heavily penalized via very small threshold p-values. As a result power will be low to detect genetic effects.

In addition to its pleasant interpretation, the FDR appears fairly robust against the effects of correlated tests in general [11], [70], [71], [74]–[77] and the correlational structure of linkage disequilibrium (LD) studies in particular [78], [79]. An intuitive explanation is that these methods use estimates of the ratio of false to total discoveries in a study. Correlated tests mainly increase the variance of these estimates. However, the FDR statistics themselves that are the means of these estimates tend to remain similar. To avoid that the FDR is controlled too conservatively we need to estimate the proportion of tests for which the null-hypothesis is true. For this purpose we used the “lowest slope” method, known to be conservatively biased toward one [80].

### Defining the QTL interval

Inbred laboratory mouse strains originated from a mixed but limited founder population [81]. Although recombination breaks up chromosomes when they are passed on to the next generation, the number of generations that occurred before inbreeding was limited. As a result current inbred mouse strains share extensive haplotypes from their founder strains, causing LD or associations among markers that are close to each other on the mouse genome. Indeed, by typing a large set of SNPs in nine inbred stains, [3] found that for most of the chromosomal regions few (e.g. two) different founder haplotypes were observed. Thus, for each of the significant markers, the QTL interval needs to be defined. LD can extend over several Mb and we therefore included wild derived inbred strains that may not share the same ancestral haplotypes in order to achieve the greatest mapping resolution.

To determine how far out meaningful LD extended from highly significant SNPs, we first calculated the *r* ^2^s between the top SNPs and every other marker in the genome. The *r* ^2^s were then ranked and the meaningful LD threshold was defined as being ranked in the one percentile. The ranking and threshold calculation was done separately for each of the top markers and showed that each marker has a different distribution of *r* ^2^ across the genome. Therefore the threshold *r* ^2^ for each marker was also different. Other rank thresholds were examined but the 0.01 level seemed to be the most useful in relation to the expected decay of LD as a function of physical distance. Physically nearby (<5 megabases) markers above the 0.01 rank threshold were considered to be in real LD and not imperfect mirrors (see below). Finally, in regions surrounding multiple associated SNPs, all marker to marker *r* ^2^s were calculated.

In addition to LD caused by the presence of common haplotypes, there is the phenomenon of markers sharing the same or highly similar pattern of genotypes across strains but that may be on different chromosomes. These ‘mirrors’ can occur by chance and the phenomenon is aggravated by the non-random mating history of the common inbred strains. These mirrors are characterized by *r* ^2^ values close or equal to 1. The problem is that mirrors will give very similar association results making it difficult to identify the exact location of the QTL. Distinguishing between meaningful LD between physically related markers caused by shared haplotypes versus mirror effect is challenging. However, we used the following procedure based on the parsimony principle to address this issue. After completing the *in silico* scan and controlling the FDR at the 0.045 level, all pairwise marker-to-marker *r* ^2^s for the significant SNPs were calculated. To determine the origin or the source of the true signal of a set of mirrors, the mirrors were first physically mapped and then the various solutions with different origins were plotted. The plot with the fewest number of origins was determined to be the most parsimonious.

## Supporting Information

Table S1 Full list of eighty-nine SNPs that satisfy a false discovery rate of 5%.
(0.14 MB DOC)

Table S2 List of the strains used in the study along with sample size and the clade assignments.
(0.06 MB DOC)
