# Supplementary material for: In Silico Whole Genome Association Scan for Murine Prepulse Inhibition
Source: PLoS One. 2009 Apr 16;4(4):e5246. doi: 10.1371/journal.pone.0005246 (PMC2666808; doi:10.1371/journal.pone.0005246)
Supplement: Table S1 — Full list of eighty-nine SNPs that satisfy a false discovery rate of 5%. (0.14 MB DOC) [file pone.0005246.s001.doc]

| **Index** | **chr** | **Mbp** | **Marker** | **p-value** |
| --- | --- | --- | --- | --- |
| 79 | 1 | 12.48415 | rs13475727 | 0.00025 |
| 86 | 1 | 13.48678 | rs3655978 | 0.000244 |
| 95 | 1 | 14.62936 | rs3713198 | 0.000244 |
| 140 | 1 | 20.99448 | rs6404446 | 4.39E-06 |
| 141 | 1 | 21.01271 | rs3716569 | 2.21E-06 |
| 142 | 1 | 21.02467 | rs4222181 | 3.33E-06 |
| 575 | 1 | 79.86539 | rs3698264 | 3.39E-05 |
| 657 | 1 | 93.31936 | rs6268443 | 4.86E-06 |
| 658 | 1 | 93.34087 | rs13475987 | 0.000167 |
| 792 | 1 | 114.4212 | mCV23695506 | 7.04E-05 |
| 802 | 1 | 115.9281 | rs3022830 | 1.78E-05 |
| 811 | 1 | 117.1431 | rs3694226 | 4.39E-06 |
| 813 | 1 | 117.3378 | rs3662732 | 1.2E-06 |
| 814 | 1 | 117.3797 | rs3674655 | 8.14E-07 |
| 816 | 1 | 117.5264 | CEL-1_117526378 | 8.14E-07 |
| 820 | 1 | 118.2364 | rs6216134 | 1.29E-05 |
| 824 | 1 | 118.8341 | rs3719973 | 1.29E-05 |
| 825 | 1 | 118.9302 | rs13476078 | 2.67E-05 |
| 845 | 1 | 122.0107 | rs13476086 | 0.000192 |
| 3468 | 3 | 110.6496 | rs3676545 | 0.000151 |
| 4264 | 4 | 61.03476 | rs3690581 | 0.000138 |
| 4609 | 4 | 108.5813 | rs13477908 | 0.000238 |
| 5261 | 5 | 42.77623 | rs13478217 | 0.000183 |
| 5262 | 5 | 42.91475 | rs6215373 | 3.27E-05 |
| 5265 | 5 | 43.27164 | mCV22331571 | 5E-05 |
| 5266 | 5 | 43.34734 | rs3669254 | 5.59E-06 |
| 5270 | 5 | 43.66881 | rs3663092 | 5.15E-06 |
| 5706 | 5 | 105.8263 | rs3656524 | 0.000238 |
| 5712 | 5 | 106.2415 | CEL-5_106241511 | 0.000244 |
| 5720 | 5 | 107.3847 | rs3725274 | 0.000244 |
| 5909 | 5 | 135.8367 | rs4225534 | 7.26E-05 |
| 5910 | 5 | 135.8367 | rs4225533 | 0.00022 |
| 5945 | 5 | 141.0981 | rs6284348 | 0.000244 |
| 5953 | 5 | 142.025 | UT_5_143.236614 | 0.000244 |
| 6106 | 6 | 16.45757 | rs13478643 | 0.000203 |
| 6204 | 6 | 32.18179 | rs13478694 | 9.08E-05 |
| 6205 | 6 | 32.18652 | rs3678887 | 9.08E-05 |
| 6212 | 6 | 32.96114 | rs13478699 | 9.07E-05 |
| 6336 | 6 | 53.55993 | rs3688920 | 0.000238 |
| 6339 | 6 | 54.13175 | rs6272774 | 0.000244 |
| 6341 | 6 | 54.22156 | rs6372575 | 0.000244 |
| 6343 | 6 | 54.49934 | gnf06.052.076 | 0.000244 |
| 6345 | 6 | 54.66279 | rs13478765 | 0.000244 |
| 6348 | 6 | 55.13244 | rs6308025 | 0.000244 |
| 6350 | 6 | 55.41075 | rs3023071 | 0.000238 |
| 6361 | 6 | 56.52803 | CEL-6_56528034 | 0.000244 |
| 6543 | 6 | 84.37375 | CEL-6_84373747 | 0.000199 |
| 7394 | 7 | 55.14884 | rs3698065 | 0.000244 |
| 8073 | 8 | 16.70108 | CEL-8_16701082 | 0.000216 |
| 8102 | 8 | 21.20699 | rs3691954 | 4.09E-05 |
| 8103 | 8 | 21.28511 | rs4140004 | 6.81E-05 |
| 10024 | 10 | 34.4106 | rs13480578 | 0.000238 |
| 10768 | 11 | 22.90135 | rs4228627 | 7.29E-05 |
| 11014 | 11 | 57.09656 | rs13481043 | 0.000184 |
| 11016 | 11 | 57.13988 | rs3702614 | 6.81E-05 |
| 11022 | 11 | 57.74496 | rs6371333 | 0.000244 |
| 11025 | 11 | 58.03108 | rs3714321 | 0.000244 |
| 11031 | 11 | 58.61899 | rs6313602 | 0.000244 |
| 11098 | 11 | 66.7845 | rs6299418 | 4.39E-06 |
| 11699 | 12 | 23.87208 | rs3685299 | 0.000244 |
| 12555 | 13 | 42.1307 | rs13481782 | 6.06E-05 |
| 12592 | 13 | 46.62418 | rs6411274 | 0.000155 |
| 12594 | 13 | 46.8105 | rs3724682 | 4.75E-05 |
| 12595 | 13 | 46.8434 | rs3670228 | 0.000117 |
| 12596 | 13 | 46.91742 | gnf13.045.507 | 0.000117 |
| 12598 | 13 | 47.17024 | rs3682400 | 0.000183 |
| 13491 | 14 | 53.76341 | rs3699179 | 0.000244 |
| 13495 | 14 | 54.79427 | rs6189337 | 0.000238 |
| 13512 | 14 | 56.52708 | rs3721918 | 0.000244 |
| 13514 | 14 | 56.72865 | rs3700859 | 0.000244 |
| 13519 | 14 | 57.34776 | rs3697645 | 0.000244 |
| 13522 | 14 | 58.14749 | rs3700249 | 0.000238 |
| 13523 | 14 | 58.16278 | rs6378901 | 0.000238 |
| 13956 | 15 | 4.403642 | rs6277526 | 0.000197 |
| 13957 | 15 | 4.736328 | rs6256742 | 0.000244 |
| 13959 | 15 | 5.009737 | rs6319377 | 0.000244 |
| 13962 | 15 | 5.475686 | rs6342976 | 0.000244 |
| 13972 | 15 | 6.457051 | rs6296430 | 0.000244 |
| 13974 | 15 | 6.759076 | rs6315163 | 0.000244 |
| 13976 | 15 | 7.313852 | rs6180855 | 0.000244 |
| 13978 | 15 | 7.623744 | rs6231675 | 0.000175 |
| 13982 | 15 | 8.23831 | rs6303661 | 0.000244 |
| 14528 | 15 | 83.2835 | CEL-15_83283496 | 0.000244 |
| 14897 | 16 | 35.12425 | rs4172689 | 0.000238 |
| 14977 | 16 | 45.139 | rs3723465 | 0.000244 |
| 14978 | 16 | 45.24729 | rs4180773 | 0.000244 |
| 16028 | 17 | 86.42377 | CEL-17_86423771 | 0.000243 |
| 16068 | 17 | 90.97095 | CEL-17_90970953 | 0.000244 |
| 16069 | 17 | 91.04428 | CEL-17_91044280 | 0.000244 |
