# Supplementary material for: In Silico Whole Genome Association Scan for Murine Prepulse Inhibition
Source: PLoS One. 2009 Apr 16;4(4):e5246. doi: 10.1371/journal.pone.0005246 (PMC2666808; doi:10.1371/journal.pone.0005246)
Supplement: Table S2 — List of the strains used in the study along with sample size and the clade assignments. (0.06 MB DOC) [file pone.0005246.s002.doc]

| Strain | Clade | N | PPI mean |
| --- | --- | --- | --- |
| 129S1/SvImJ | 5 | 20 | 1.48 |
| A/J | 1 | 20 | 2.17 |
| AKR/J | 1 | 20 | 1.30 |
| BALB/cJ | 1 | 28 | 1.89 |
| BTBR_T+_tf/J | 5 | 20 | 1.77 |
| BUB/BnJ | 2 | 20 | 1.83 |
| C3H/HeJ | 1 | 20 | 2.34 |
| C57BL/10J | 4 | 20 | 1.30 |
| C57BL/6J | 4 | 21 | 1.88 |
| C57BR/cdJ | 4 | 20 | 2.43 |
| C57L/J | 4 | 20 | 3.16 |
| C58/J | 4 | 20 | 1.33 |
| CAST/EiJ | 7 | 20 | 2.23 |
| CBA/J | 1 | 19 | 1.71 |
| CE/J | 1 | 20 | 1.37 |
| DBA/2J | 6 | 20 | 2.43 |
| FVB/NJ | 2 | 20 | 1.67 |
| I/LnJ | 6 | 20 | 2.26 |
| KK/HlJ | 3 | 20 | 1.74 |
| LP/J | 5 | 20 | 1.47 |
| MA/MyJ | 2 | 20 | 1.66 |
| MOLF/EiJ | 7 | 20 | 2.56 |
| MSM/Ms | 7 | 20 | 2.23 |
| NOD/LtJ | 2 | 20 | 2.42 |
| NON/LtJ | 3 | 20 | 2.37 |
| NZB/BlNJ | 3 | 20 | 2.08 |
| NZW/LacJ | 3 | 20 | 1.46 |
| PERA/EiJ | 7 | 20 | 2.19 |
| PL/J | 1 | 20 | 1.44 |
| PWK/PhJ | 7 | 18 | 2.04 |
| RIIIS/J | 2 | 20 | 1.47 |
| SEA/GnJ | 1 | 24 | 1.88 |
| SJL/J | 2 | 20 | 2.57 |
| SM/J | 6 | 20 | 1.23 |
| SPRET/EiJ | 7 | 20 | 1.60 |
| SWR/J | 2 | 20 | 1.49 |
| WSB/EiJ | 7 | 15 | 2.04 |
